# Supplementary material for: Modelling the developmental spliceosomal craniofacial disorder Burn-McKeown syndrome using induced pluripotent stem cells
Source: PLoS One. 2020 Jul 31;15(7):e0233582. doi: 10.1371/journal.pone.0233582 (PMC7394406; doi:10.1371/journal.pone.0233582)
Supplement: S2 File — (PDF) [file pone.0233582.s003.pdf]

Supporting Information

Modelling the developmental spliceosomal craniofacial disorder Burn-McKeown syndrome using induced pluripotent stem cells

Katherine A. Wood[1,2], Charlie F. Rowlands[1,2], Huw B. Thomas[1], Steven Woods[3], Julieta O'Flaherty[3], Sofia Douzgou[1,2], Susan J. Kimber[3], William G. Newman[1,2], Raymond T. O'Keefe[1*]*

[1]Division of Evolution and Genomic Sciences, School of Biological Sciences, Faculty of Biology, Medicine and Health, The University of Manchester, UK

[2]Manchester Centre for Genomic Medicine, Manchester University NHS Foundation Trust, Manchester Academic Health Science Centre, Manchester, UK

[3]Division of Cell Matrix Biology and Regenerative Medicine, School of Biology, Faculty of Biology, Medicine and Health, University of Manchester, Manchester, UK

*To whom correspondence should be addressed.

Email: william.newman@manchester.ac.uk; rokeefe@manchester.ac.uk

SUPPLEMENTARY FIGURES S1– S10

S1 FIG

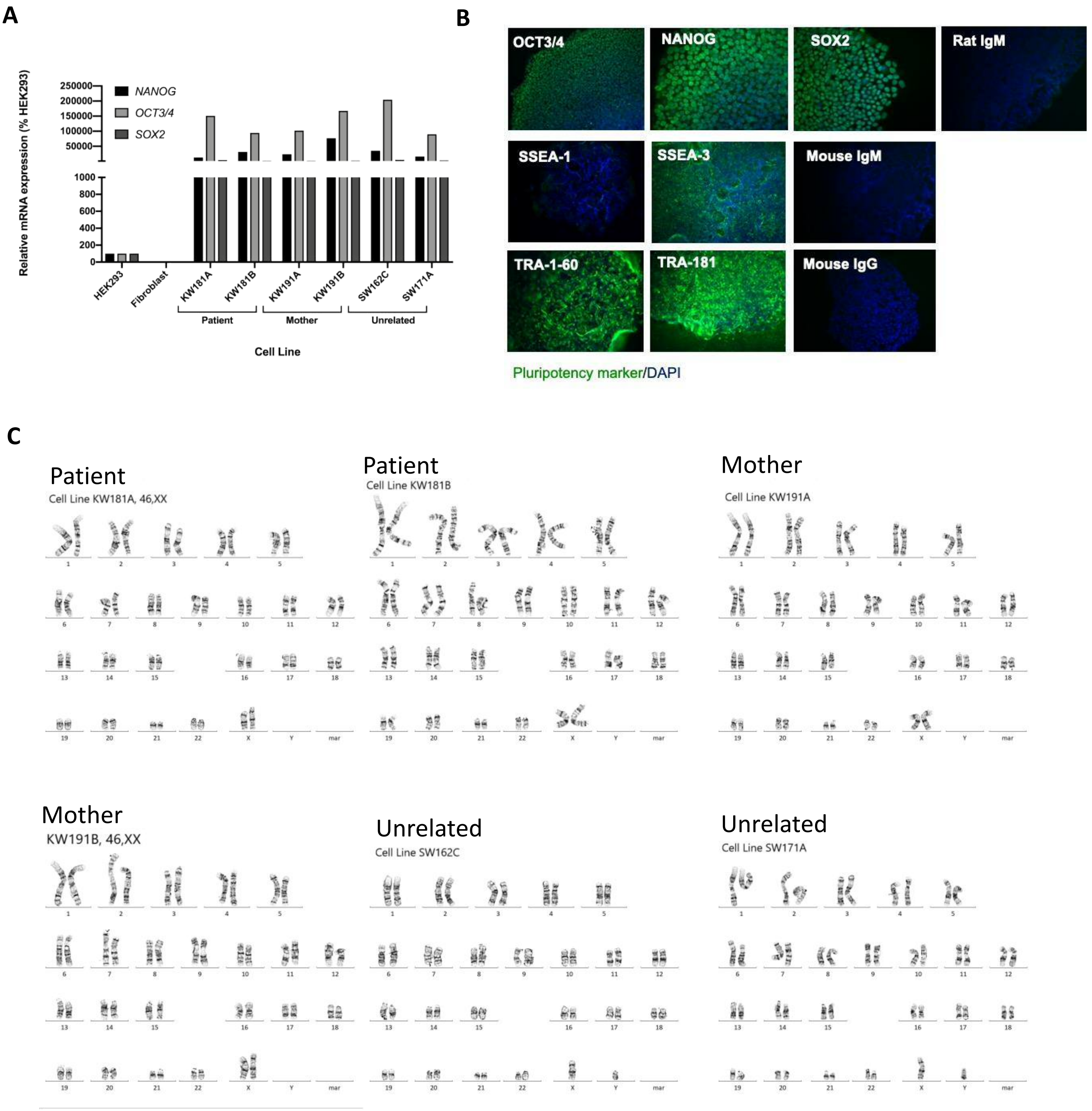

D

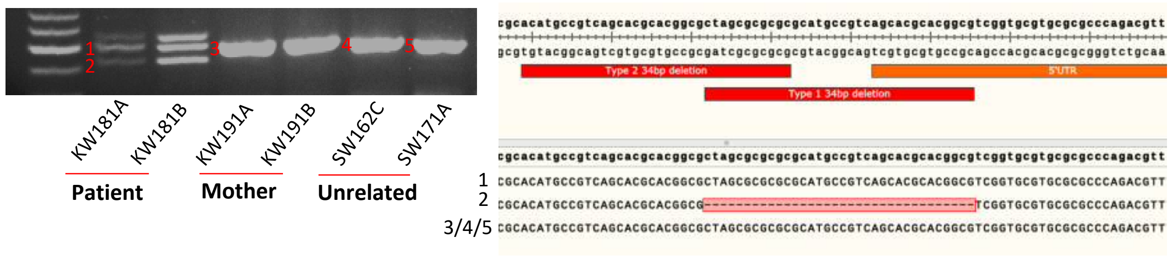

E

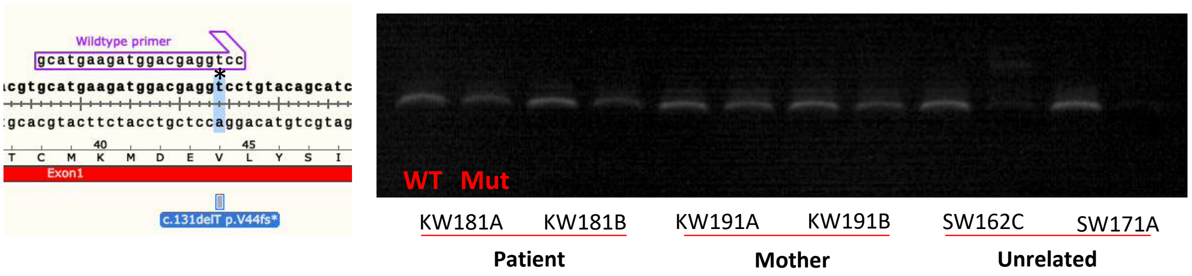

S1 Fig: Characteristics of iPSCs. A) Relative pluripotency marker gene (*OCT3/4*, *SOX2*, *NANOG*) mRNA expression levels in iPSC lines (KW181A, KW181B, KW191A, KW191B, SW162C, SW171A) compared to HEK293 cells and fibroblasts, determined using qPCR of cDNA from each cell line. Graphs were obtained using the $\Delta\Delta C_T$ method with *ACTB* as the endogenous reference gene. B) Immunofluorescence staining for pluripotency markers. Representative positive immunofluorescence staining in SW171A iPSCs for pluripotency markers OCT3/4, SOX2, NANOG, SSEA-3, TRA-1-60 and TRA-1-81 (green), and negative immunofluorescence staining for the early differentiation marker SSEA-1, and DAPI nuclear staining shown in blue. Isotype controls for rat IgM, mouse IgM and mouse IgG are also presented. C) Karyograms for all six iPSC lines used in this study showing normal karyotypes in all cases. D) Genotyping of type 1 34bp *TXNL4A* promoter deletion in patient (KW181A and KW181B), mother (KW191A and KW191B) and unrelated control (SW162C and SW171A) iPSC lines by PCR with genomic DNA extracted from cell lines and primers proximal to the deleted region in the *TXNL4A* promoter. PCR products were separated by agarose gel electrophoresis and purified, and the identities confirmed by Sanger sequences as shown. The highest molecular weight (MW) band in the patient did not sequence. E) Genotyping of the chr18: g.77,748,262delA, RefSeq NM_006701.2; c.131delT (p.Val44Alafs*48) (GRCh37/hg19) (exon 1, 1bp deletion) in iPSC lines. Genomic DNA extracted from the cell lines was amplified in two PCR reactions, one with the wildtype forward primer as shown and one with a mutant forward primer with the ‘t’ base indicated by a * deleted. PCR products were separated by agarose gel electrophoresis. Cell lines heterozygous for the mutation amplify with both primer sets while cell lines homozygous for the wildtype sequence or the mutant sequence only amplify with the wildtype or mutant primer set respectively.

S2 FIG

A

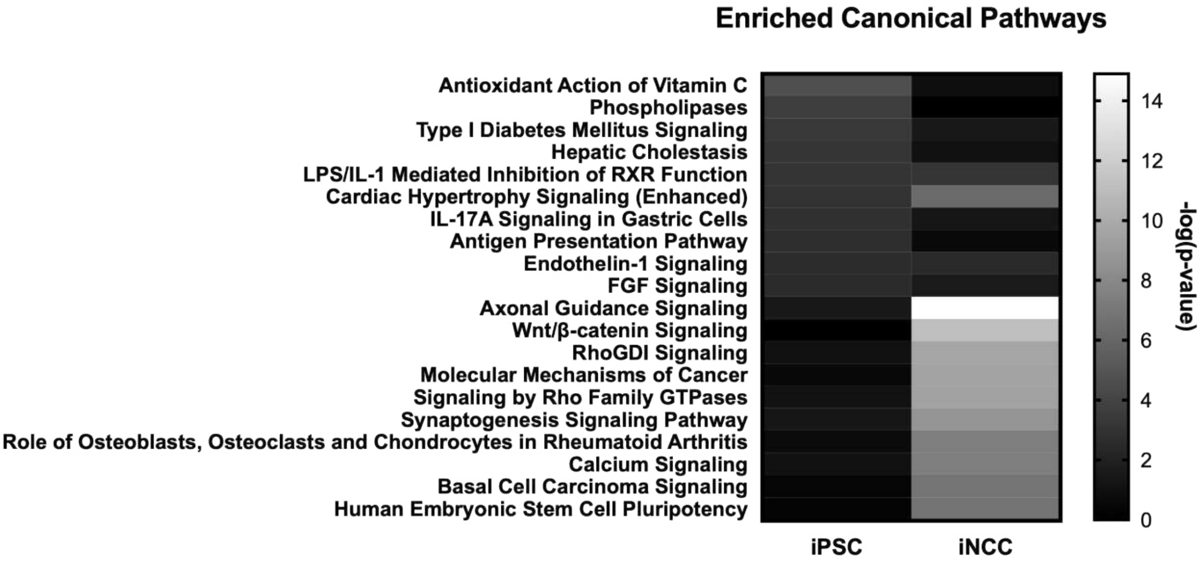
Enriched Canonical Pathways
Antioxidant Action of Vitamin C
Phospholipases
Type I Diabetes Mellitus Signaling
Hepatic Cholestasis
LPS/IL-1 Mediated Inhibition of RXR Function
Cardiac Hypertrophy Signaling (Enhanced)
IL-17A Signaling in Gastric Cells
Antigen Presentation Pathway
Endothelin-1 Signaling
FGF Signaling
Axonal Guidance Signaling
Wnt/β-catenin Signaling
RhoGDI Signaling
Molecular Mechanisms of Cancer
Signaling by Rho Family GTPases
Synaptogenesis Signaling Pathway
Role of Osteoblasts, Osteoclasts and Chondrocytes in Rheumatoid Arthritis
Calcium Signaling
Basal Cell Carcinoma Signaling
Human Embryonic Stem Cell Pluripotency
iPSC
iNCC
14
12
10
8
6
4
2
0
-log(p-value)

B

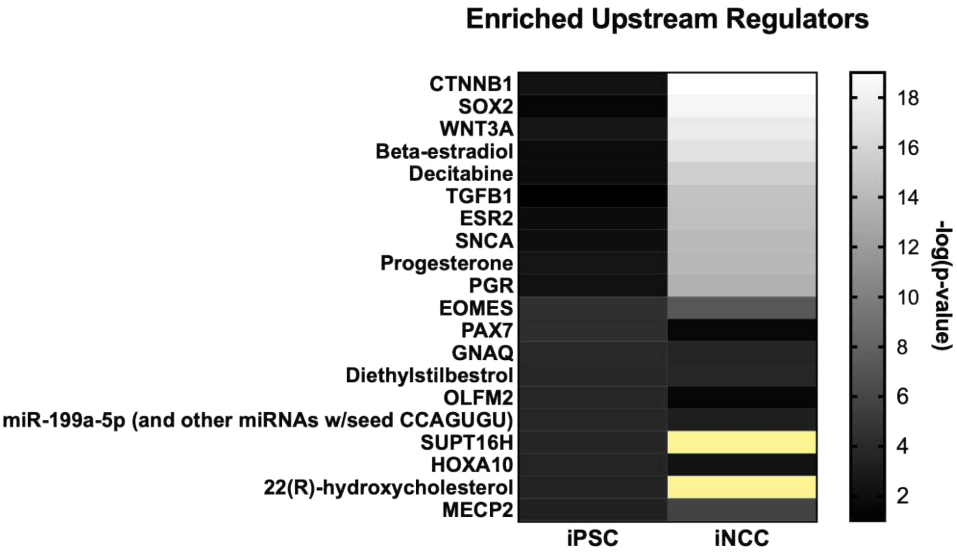
Enriched Upstream Regulators
CTNNB1
SOX2
WNT3A
Beta-estradiol
Decitabine
TGFB1
ESR2
SNCA
Progesterone
PGR
EOMES
PAX7
GNAQ
Diethylstilbestrol
OLFM2
miR-199a-5p (and other miRNAs w/seed CCAGUGU)
SUPT16H
HOXA10
22(R)-hydroxycholesterol
MECP2
iPSC
iNCC
18
16
14
12
10
8
6
4
2
-log(p-value)

C

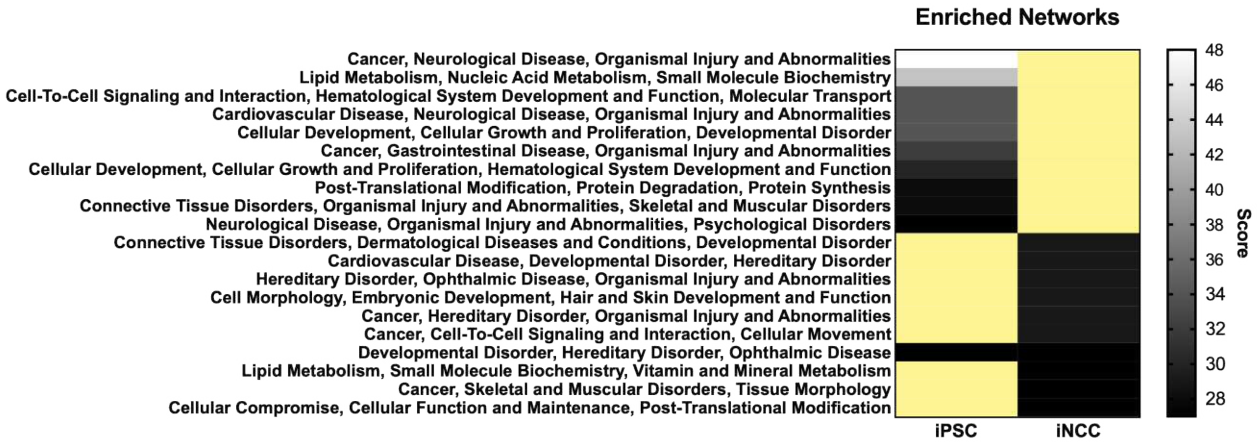

D

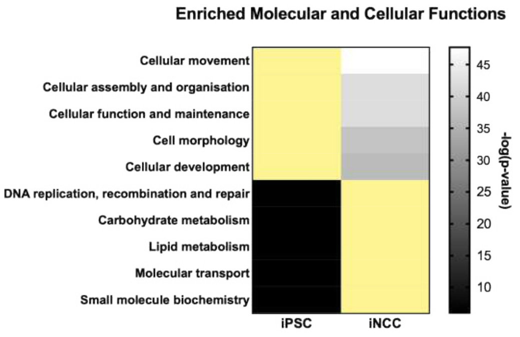

E

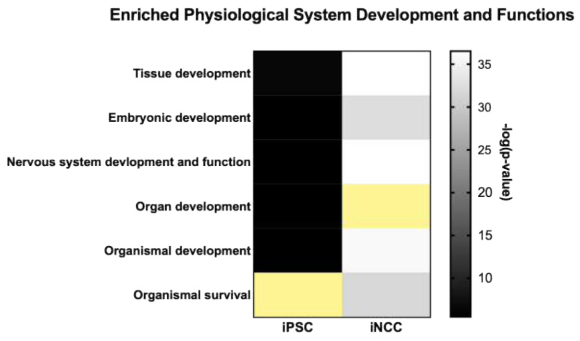

S2 Fig: Top enriched A) canonical pathways, B) networks, C) upstream regulators, D) molecular and cellular functions, E) physiological system development and functions, associated with the differentially expressed genes (DEGs) in pooled patient iPSCs or iNCCs compared to pooled maternal and unrelated control iPSCs or iNCCs, obtained using IPA. Pale yellow indicates no enrichment for the particular term in that cell line.

S3 FIG

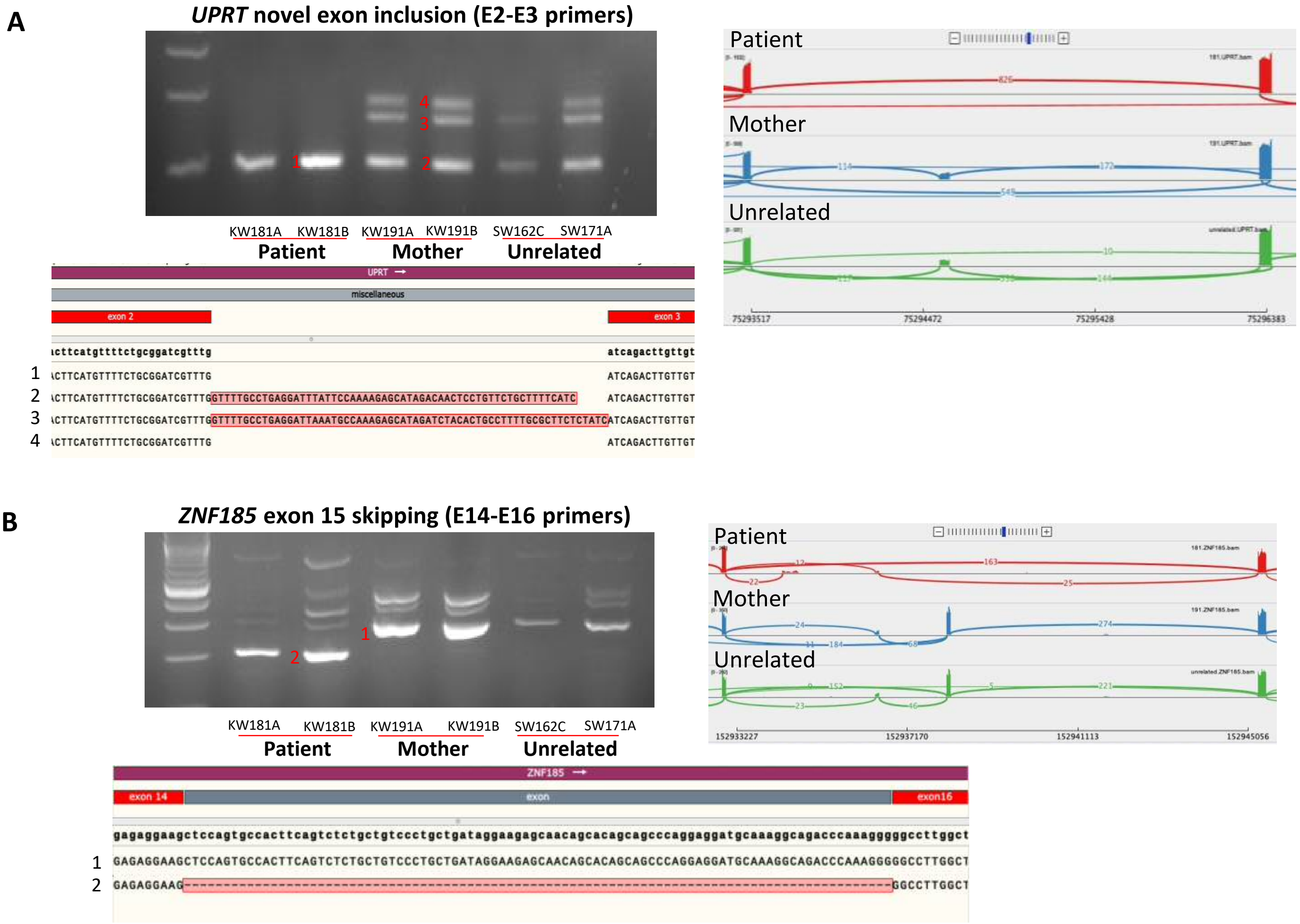

S3 Fig: Validation of alternative splicing events in A) *UPRT* and B) *ZNF185* as identified by rMATS in patient iPSCs (KW181A, KW181B) compared to mother (KW191A, KW191B) and unrelated control (SW162C, SW171A) iPSCs. For both genes, Sashimi plots identified inclusion of an exon in mother and unrelated iPSC lines not apparent in patient lines. RT-PCR reactions were performed using primers targeting the exons flanking the novel exons with cDNA generated from the RNA extracted from iPSCs and used in RNA-Seq experiments. PCR products were separated by agarose gel electrophoresis, and the products indicated by red numbers were purified and the corresponding sequences (confirmed by Sanger sequencing) are shown below.

S4 FIG

A

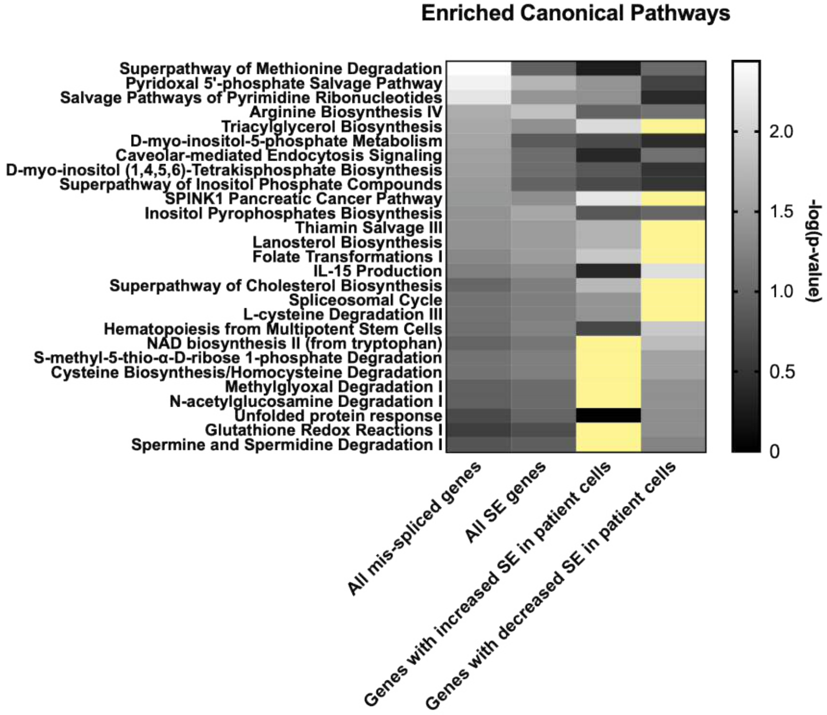
Enriched Canonical Pathways
Superpathway of Methionine Degradation
Pyridoxal 5'-phosphate Salvage Pathway
Salvage Pathways of Pyrimidine Ribonucleotides
Arginine Biosynthesis IV
Triacylglycerol Biosynthesis
D-myo-inositol-5-phosphate Metabolism
Caveolar-mediated Endocytosis Signaling
D-myo-inositol (1,4,5,6)-Tetrakisphosphate Biosynthesis
Superpathway of Inositol Phosphate Compounds
SPINK1 Pancreatic Cancer Pathway
Inositol Pyrophosphates Biosynthesis
Thiamin Salvage III
Lanosterol Biosynthesis
Folate Transformations I
IL-15 Production
Superpathway of Cholesterol Biosynthesis
Spliceosomal Cycle
L-cysteine Degradation III
Hematopoiesis from Multipotent Stem Cells
NAD biosynthesis II (from tryptophan)
S-methyl-5-thio-α-D-ribose 1-phosphate Degradation
Cysteine Biosynthesis/Homocysteine Degradation
Methylglyoxal Degradation I
N-acetylglucosamine Degradation I
Unfolded protein response
Glutathione Redox Reactions I
Spermine and Spermidine Degradation I
2.0
1.5
1.0
0.5
0
-log(p-value)
All mis-spliced genes
All SE genes
Genes with increased SE in patient cells
Genes with decreased SE in patient cells

B

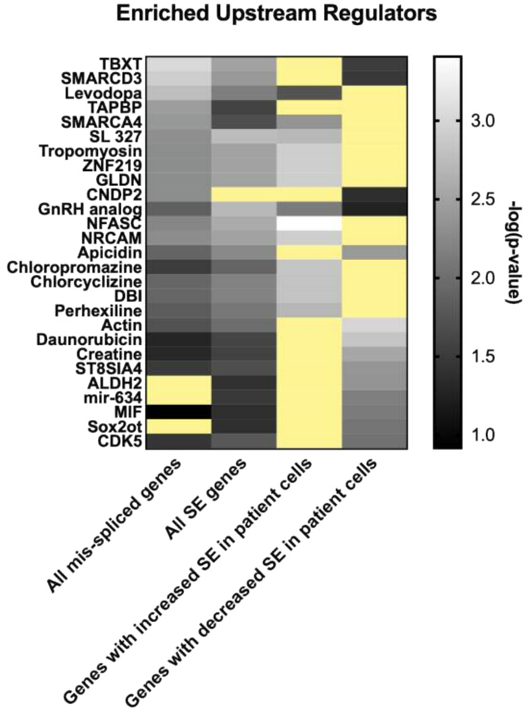
Enriched Upstream Regulators
TBXT
SMARCD3
Levodopa
TAPBP
SMARCA4
SL 327
Tropomyosin
ZNF219
GLDN
CNDP2
GnRH analog
NFASC
NRCAM
Apicidin
Chloropromazine
Chlorcyclizine
DBI
Perhexiline
Actin
Daunorubicin
Creatine
ST8SIA4
ALDH2
mir-634
MIF
Sox2ot
CDK5
3.0
2.5
2.0
1.5
1.0
-log(p-value)
All mis-spliced genes
All SE genes
Genes with increased SE in patient cells
Genes with decreased SE in patient cells

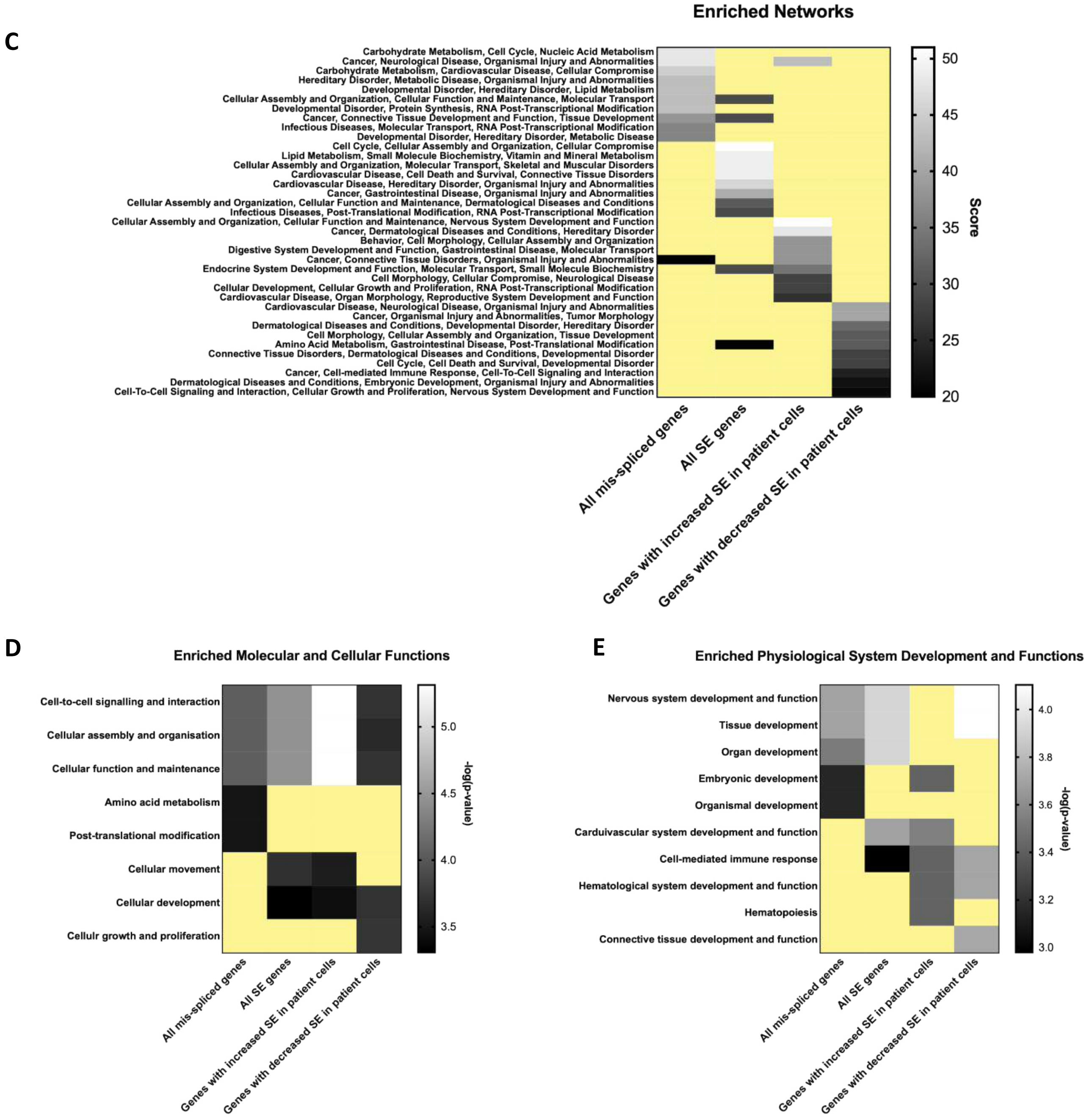

S4 Fig: Top enriched A) canonical pathways, B) networks, C) upstream regulators, D) molecular and cellular functions, E) physiological system development and functions, associated with all the mis-spliced genes in pooled patient iPSCs compared to pooled maternal and unrelated control iPSCs, all genes showing altered exon skipping (SE) in pooled patient iPSCs compared to pooled maternal and unrelated control iPSCs, genes showing increased SE and genes showing decreased SE in pooled patient iPSCs compared to pooled maternal and unrelated control iPSCs, obtained using IPA. Pale yellow indicates no enrichment for the particular term in that cell line.

S5 FIG

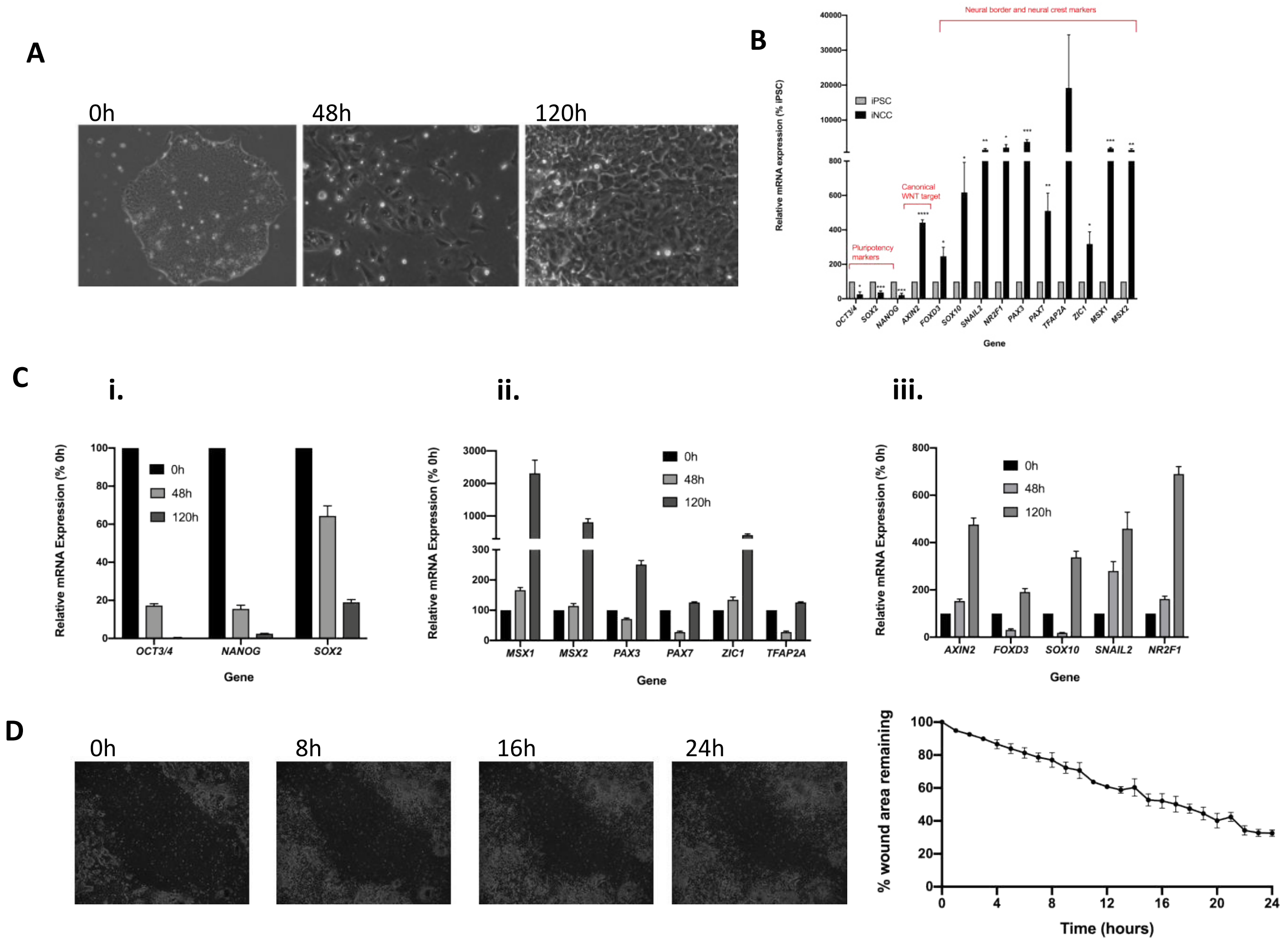

S5 Fig: Differentiation of SW171A control iPSCs to iNCCs. A) Key stages in differentiation of SW171A unrelated control iPSCs to iNCCs. Phase contrast microscopy showing the changing morphology of cells at time points 0h, 48h and 120h in the differentiation protocol described by Leung et al., (2016). B) Key marker gene expression in SW171A cells pre- and post-differentiation to iNCCs, obtained by qPCR on cDNA generated from RNA isolated from the cells at 0h and 120h of the protocol. Relative gene expression was calculated using the $\Delta\Delta C_T$ method with *ACTB* as a stable endogenous reference gene. n = 4. C) Expression of i) key pluripotency marker genes, ii) neural border and iii) *AXIN2* and neural crest marker genes in SW171A control cells at 0h, 48h and 120h during the differentiation protocol, obtained by qPCR on cDNA generated from RNA isolated from the cells at the specified time points. Relative gene expression was calculated using the $\Delta\Delta C_T$ method with *ACTB* as a stable endogenous reference gene. n = 3. D) Migration of differentiated SW171A iNCCs determined using a wound healing assay. Representative phase-contrast images taken immediately and at 8h, 16h and 24h after wounding. The wound area at each time point was calculated using the MRI wound healing plugin in the Fiji image analysis tool. n = 2.

S6 FIG

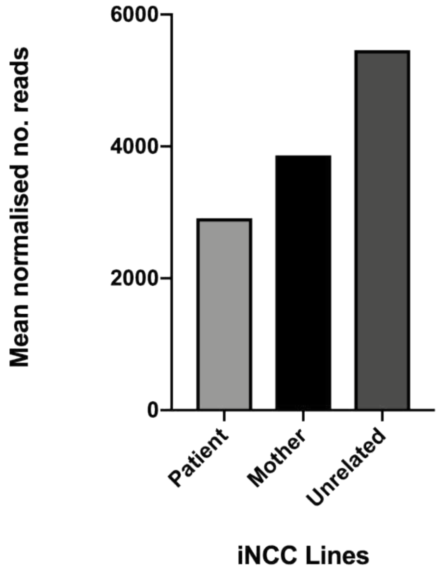

S6 Fig: Mean normalised read counts for *TXNL4A* in pooled patient, mother and unrelated control iNCCs from RNA-Seq data from iNCCs.

S7 FIG

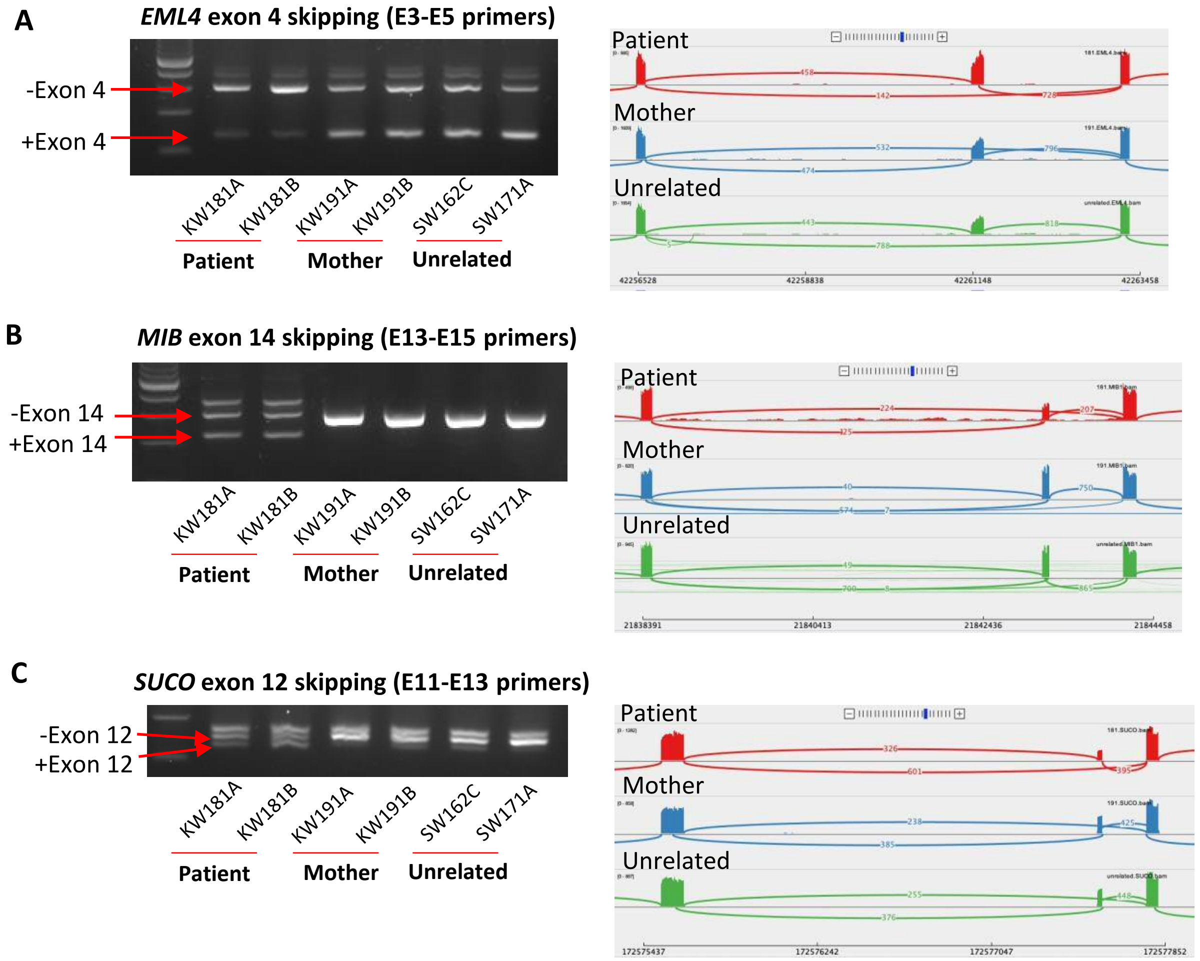

S7 Fig: Validation of alternative splicing events in A) *EML4*, B) *MIB* and C) *SUCO* as identified by rMATS in pooled patient (KW181A and KW181B) iNCCs compared to pooled mother (KW191A and KW191B) and unrelated control (SW162C and SW171A) iNCCs. For all genes, Sashimi plots identified altered splicing events. RT-PCR reactions were performed using primers targeting the exons flanking the novel exons with cDNA generated from the RNA extracted from iNCCs and used in RNA-Seq experiments. PCR products were separated by agarose gel electrophoresis

S8 FIG

A

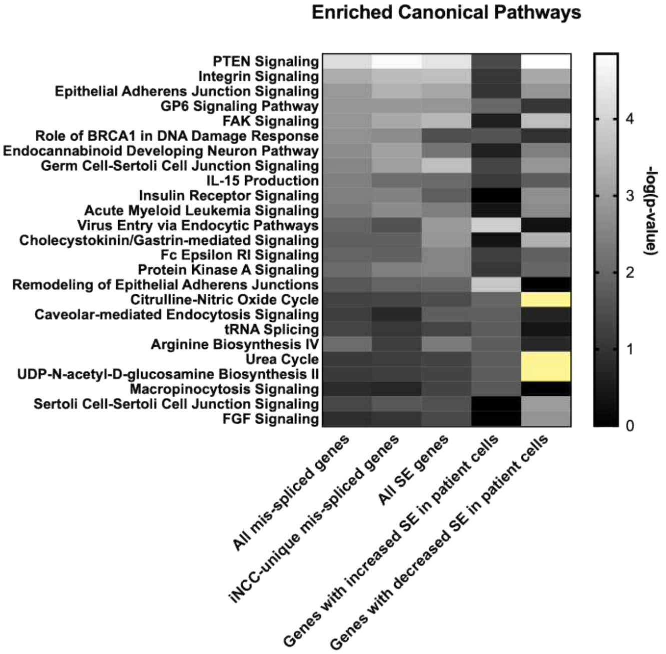
Enriched Canonical Pathways
PTEN Signaling
Integrin Signaling
Epithelial Adherens Junction Signaling
GP6 Signaling Pathway
FAK Signaling
Role of BRCA1 in DNA Damage Response
Endocannabinoid Developing Neuron Pathway
Germ Cell-Sertoli Cell Junction Signaling
IL-15 Production
Insulin Receptor Signaling
Acute Myeloid Leukemia Signaling
Virus Entry via Endocytic Pathways
Cholecystokinin/Gastrin-mediated Signaling
Fc Epsilon RI Signaling
Protein Kinase A Signaling
Remodeling of Epithelial Adherens Junctions
Citrulline-Nitric Oxide Cycle
Caveolar-mediated Endocytosis Signaling
tRNA Splicing
Arginine Biosynthesis IV
Urea Cycle
UDP-N-acetyl-D-glucosamine Biosynthesis II
Macropinocytosis Signaling
Sertoli Cell-Sertoli Cell Junction Signaling
FGF Signaling
All mis-spliced genes
iNCC-unique mis-spliced genes
All SE genes
Genes with increased SE in patient cells
Genes with decreased SE in patient cells
-log(p-value)
0
1
2
3
4

B

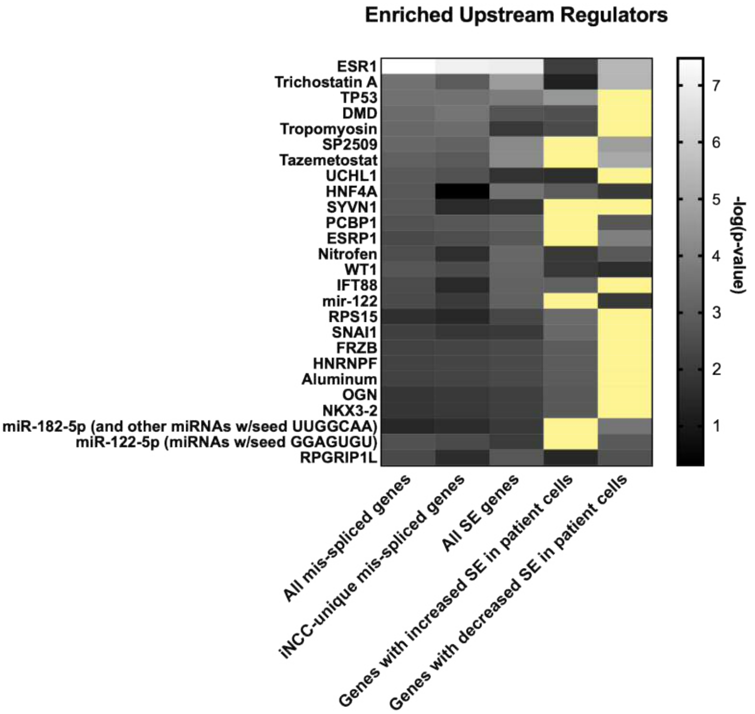
Enriched Upstream Regulators
ESR1
Trichostatin A
TP53
DMD
Tropomyosin
SP2509
Tazemetostat
UCHL1
HNF4A
SYVN1
PCBP1
ESRP1
Nitrofen
WT1
IFT88
mir-122
RPS15
SNAI1
FRZB
HNRNPF
Aluminum
OGN
NKX3-2
miR-182-5p (and other miRNAs w/seed UUGGCAA)
miR-122-5p (miRNAs w/seed GGAGUGU)
RPGRIP1L
All mis-spliced genes
iNCC-unique mis-spliced genes
All SE genes
Genes with increased SE in patient cells
Genes with decreased SE in patient cells
-log(p-value)
1
2
3
4
5
6
7

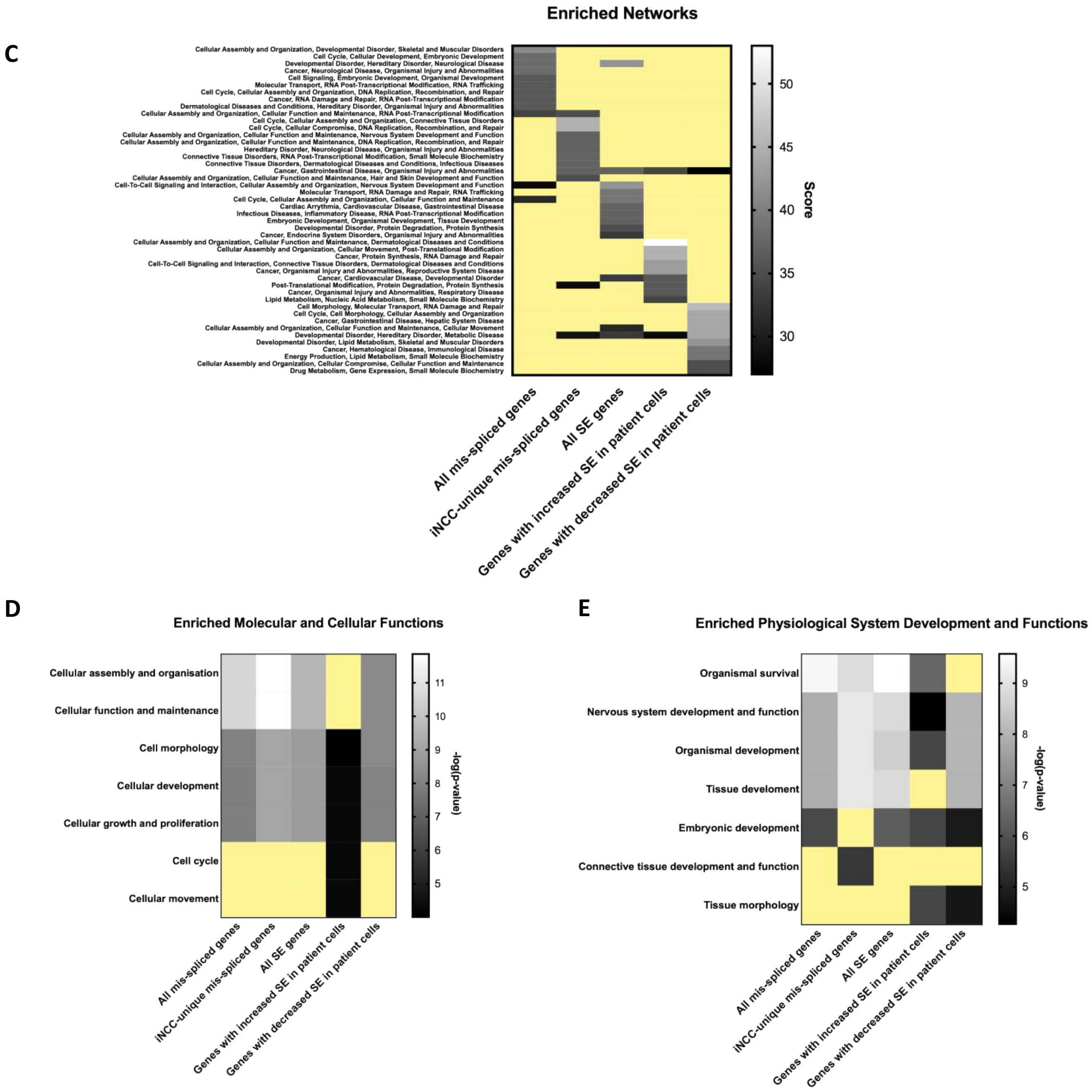

S8 Fig: Top enriched A) canonical pathways, B) networks, C) upstream regulators, D) molecular and cellular functions, E) physiological system development and functions, associated with all the mis-spliced genes in pooled patient iPSCs compared to pooled maternal and unrelated control iNCCss, all the genes which are uniquely mis-spliced between patient and pooled maternal and control cells in the iNCC RNA-Seq data and not in the iPSC RNA-Seq data, all genes showing altered exon skipping (SE) in pooled patient iNCCs compared to pooled maternal and unrelated control iNCCs, genes showing increased SE and genes showing decreased SE in pooled patient iNCCs s compared to pooled maternal and unrelated control iNCCs, obtained using IPA. Pale yellow indicates no enrichment for the particular term in that cell line.

S9 FIG

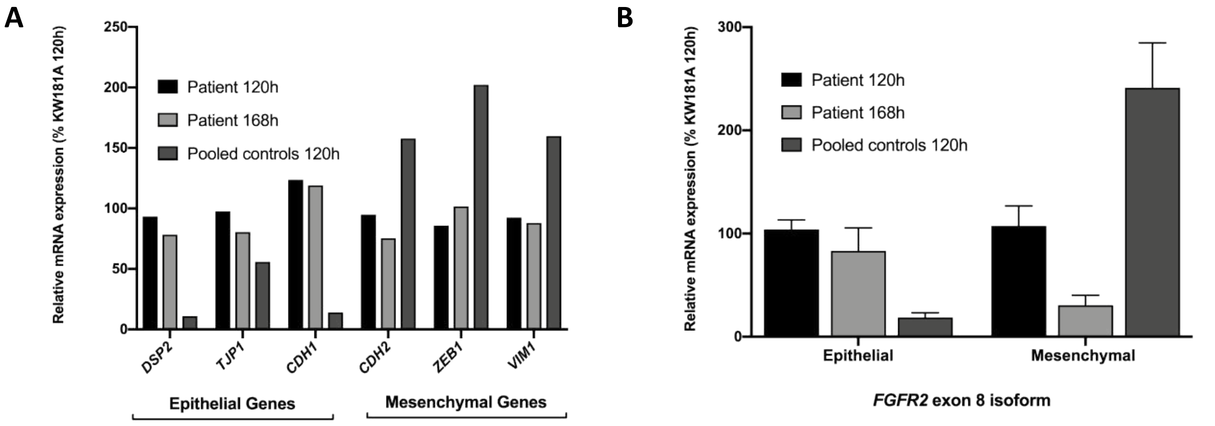

S9 Fig: EMT marker gene expression and *FGFR2* splicing following an extended differentiation period. A) epithelial and mesenchymal marker gene expression and B) *FGFR2* exon 8 splicing in 120h and 168h differentiated patient cells compared to 120h mother and unrelated (pooled control) cells, obtained using qPCR. qPCR experiments were performed on cDNA generated from RNA isolated from cell lines at the time points indicated. Relative gene expression was calculated using the $\Delta\Delta C_T$ method with *ACTB* as a stable endogenous reference gene. n = 2.

S10 FIG

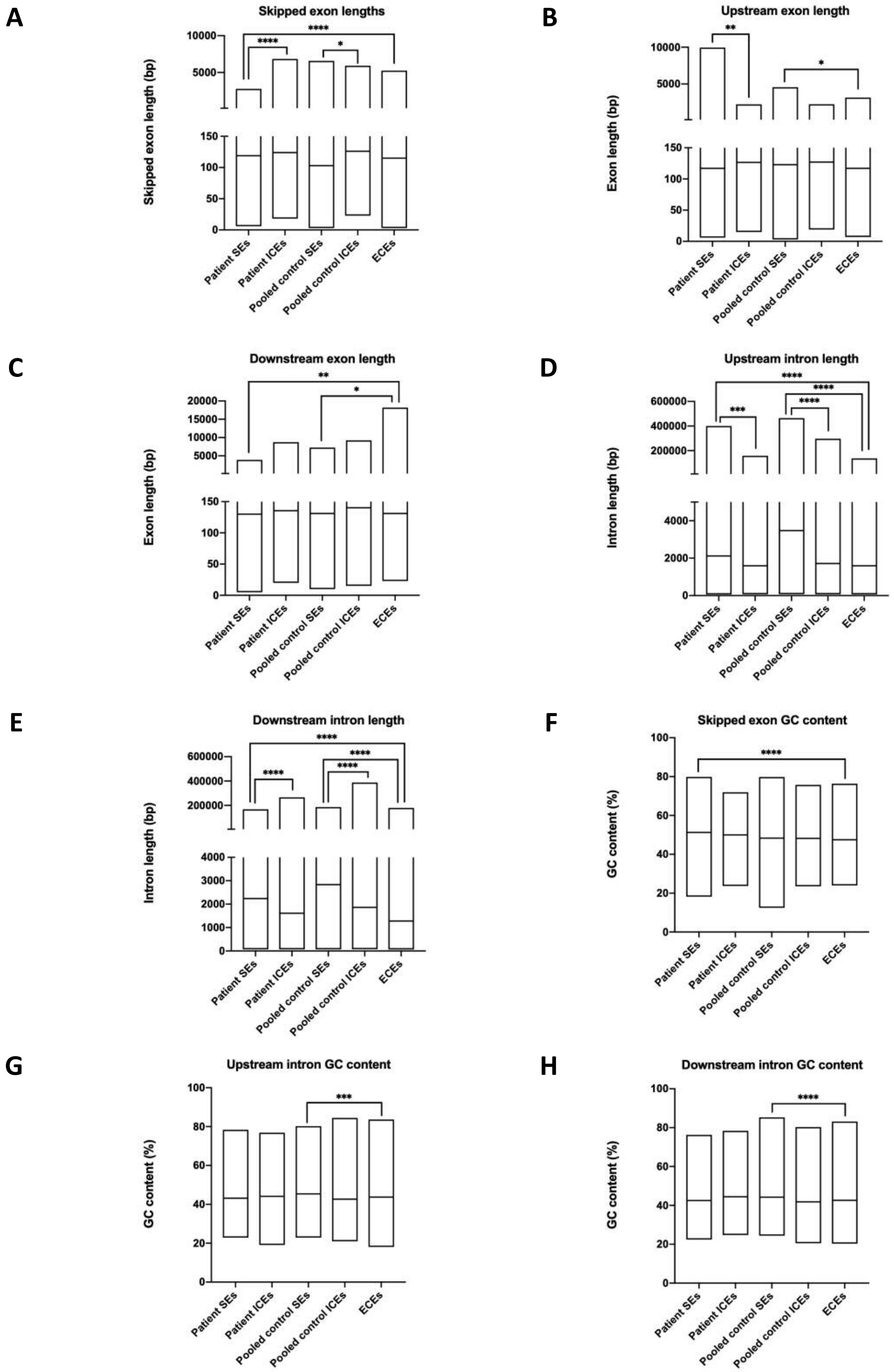
A
Skipped exon lengths
Skipped exon length (bp)
B
Upstream exon length
Exon length (bp)
C
Downstream exon length
Exon length (bp)
D
Upstream intron length
Intron length (bp)
E
Downstream intron length
Intron length (bp)
F
Skipped exon GC content
GC content (%)
G
Upstream intron GC content
GC content (%)
H
Downstream intron GC content
GC content (%)
Patient SEs
Patient ICEs
Pooled control SEs
Pooled control ICEs
ECEs

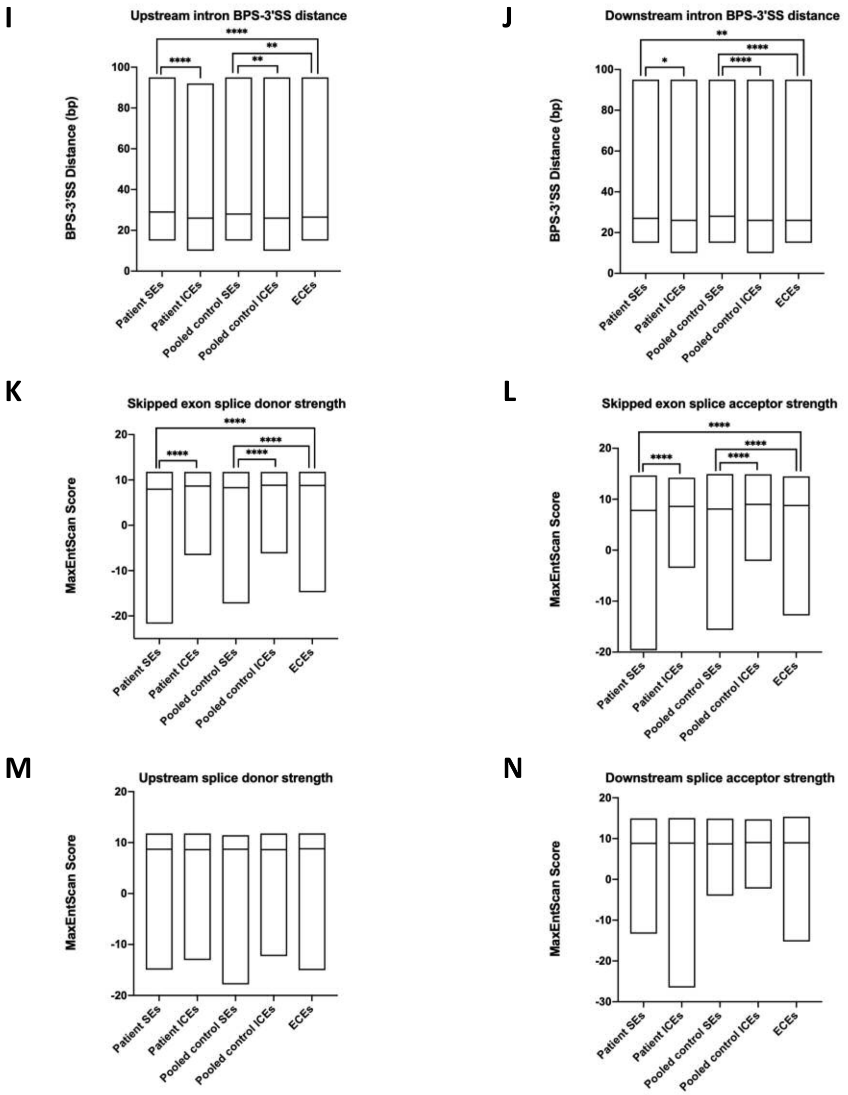

S10 Fig: Physical properties of exons more skipped (patient SE) in pooled patient iNCCs and exons less skipped (pooled control SE) in pooled patient iNCCs compared to pooled mother and unrelated control iNCCs as determined by rMATS. Physical properties were compared to internal control exons (ICEs) from the same gene containing the differential SE event, and to external control exons (ECEs) from highly expressed but not differentially spliced genes between patient and pooled control iNCCs in the RNA-Seq dataset. Properties investigated for both types of skipped exon were: A) skipped exon lengths; B) upstream exon lengths; C) downstream exon lengths; D) upstream intron lengths; E) downstream intron lengths; F) skipped exon GC content; G) upstream intron GC content; H) downstream intron GC content; I) upstream intron branch point to 3’ splice site (BPS-3’SS) distance; J) downstream intron BPS-3’SS distance; K) skipped exon splice donor strengths; L) skipped exon splice acceptor strengths; M) upstream splice donor strengths; N) downstream splice donor strengths. * p-value < 0.05, ** p-value < 0.01, *** p-value < 0.001, **** p-value < 0.0001.

SUPPLEMENTARY TABLES S1 – S7

Primer function	Target	Forward primer sequence (5' – 3')	Reverse primer sequence (5' – 3')
Genotyping	*TXNL4A* promoter deletion region	GACAGCTGCTATGACGGAACC	CAAACTCCGCTGGGACTGC
Genotyping	*TXNL4A* E1 1bp deletion (WT)	GCATGAAGATGGACGAGGTCC	TCCTCGGGGAACAGCTCG
Genotyping	*TXNL4A* E1 1bp deletion (Mut)	GCATGAAGATGGACGAGGCC	TCCTCGGGGAACAGCTCG
qPCR	*ACTB*	GTGGATCAGCAAGCAGGAGT	GTAACAACGCATCTCATATTTGGAA
qPCR	*TXNL4A*	GGACTGGCAACAACAACAAGA	AGCGGTACTTGGTGGAGTAGT
qPCR	*OCT3/4*	CTGGGTTGATCTCGGACCT	CCATCGGAGTTGCTCTCCA
qPCR	*NANOG*	TTTGTGGGCCTGAAGAAAACT	AGGGCTGTCCTGAATAAGCAG
qPCR	*SOX2*	GCCCGAGTGGAAACTTTTGTCG	GGCAGCGTGTACTTATCCTTCT
qPCR	*AXIN2*	AGTCAGCAGAGGGACAGGAA	AGCTCTGAGCCTTCAGCATC
qPCR	*FOXD3*	GCATCTGCGAGTTCATCAGC	CGTTGAGTGAGAGGTTGTGG
qPCR	*SOX10*	CTCTGGAGGCTGCTGAA	TGGGCTGGTACTTGTAGTC
qPCR	*SNAI2*	CAGACCCTGGTTGCTTCAAG	GAGCCCTCAGATTTGACCTG
qPCR	*NR2F1*	CGAGTACAGCTGCCTCAAAG	TACTGGCTCCTCACGTACTC
qPCR	*PAX3*	AATTACTCAAGGACGCGGTC	TTCTTCTCGCTTTCCTCTGC
qPCR	*PAX7*	TGACAGCTTCATGAATCCGG	GATGGAGAAGTCAGCCTGTG
qPCR	*TFAP2A*	GATCCTCGCAGGGACTACAG	TACCCGGGTCTTCTACATGC
qPCR	*ZIC2*	GTCCTACACGCATCCCAGTT	GCGATAAGGAGCTTGTGGTC
qPCR	*MSX1*	CTGCACCCTCCGCAAACACA	AGGCTGAGCGAGCTGGAGAA
qPCR	*MSX2*	CGGTCAAGTCGGAAAATTCA	GAGGAGCTGGGATGTGGTAA
qPCR	*FGFR2* E7 – E8 (NM_022970)	ATCCAGTGGATCAAGCACGTGG	GGTCACATTGAACAGAGCCAGC
qPCR	*FGFR2* E7 – E8 (NM_000141)	ATCCAGTGGATCAAGCACGTGG	CAGAACTGTCAACCATGCAGAGTG
qPCR	*DSP*	GCAGGATGTACTATTCTCGGC	CCTGGATGGTGTTCTGGTTCT
qPCR	*TJP1*	CAACATACAGTGACGCTTCACA	CACTATTGACGTTTCCCCACTC
qPCR	*CDH1*	CGAGAGCTACACGTTCACGG	GGGTGTCGAGGGAAAAATAGG
qPCR	*CDH2*	AGCCAACCTTAACTGAGGAGT	GGCAAGTTGATTGGAGGGATG
qPCR	*VIM*	AGTCCACTGAGTACCGGAGAC	CATTTCACGCATCTGGCGTTC
qPCR	*ZEB1*	GATGATGAATGCGAGTCAGATGC	ACAGCAGTGTCTTGTTGTTGT
qPCR/RT-PCR	*TCF7L2* E4 – E5	GCAGCACACATTACTCTGCG	TGCCTGGACATCAAGCAGTGG
qPCR/RT-PCR	*TCF7L2* E3 – E5	GCCAAGAGGCAAGATGGAGG	TGCCTGGACATCAAGCAGTGG

RT-PCR	*UPRT* E2 – E3	ACAGCCAGTAGAGGTGACTTC	TGGAGTGGTCACCATGCATTC
RT-PCR	*ZNF185* E14 – E16	AGTTGAGTGATGGCAATGTGG	GGATCTGCAGGTTGCTGAGC
RT-PCR	*EML4* E3 – E5	CAAGTCATACCAGTGCTGTCTC	CGACAATTTATTACGGCTATCATC
RT-PCR	*MIB* E13 – E15	CCAAGACCATGGATTGTGGATG	CTTGCATATCTTGGAGCTGACG
RT-PCR	*SUCO* E11 – E13	TTCATGGTAGAGATGAGCGG	TACCCTTATAAGGCTTAATGGAC

S1 Table: Applications and sequences of primers. E = exon, WT = wildtype, Mut = mutant.

**Target protein**	**Supplier, cat. number**	**Dilution**	**Intracellular or cell surface**
OCT3/4	BD Biosciences, 611202	1:100	Intracellular
NANOG	Cell Signalling Technologies, 4903	1:200	Intracellular
SOX2	Cell Signalling Technologies, 3579	1:400	Intracellular
SSEA-1	R&D Systems, MAB2155	1:400	Cell surface
SSEA-2	R&D Systems, MAB1434	1:200	Cell surface
SSEA-4	R&D Systems, MAB1435	1:200	Cell surface
TRA-160	Abcam, ab16288	1:200	Cell surface
TRA-181	Abcam, ab16289	1:200	Cell surface
Rabbit IgG	R&D systems, AB-105C	1:400	NA
Mouse IgG (serotype control)	R&D Systems, MAB002	1:200	NA
Mouse IgM (serotype control)	Abcam, ab18401	1:200	NA
Rat IgM (serotype control)	Abcam, ab35768	1:200	NA
Alexa-Fluor secondary anti-mouse, anti-rabbit and anti-rat antibodies (Alex-Fluor-488 and Alexa-Fluor-594)	Invitrogen	1:200	NA

S2 Table: Antibodies used for immunofluorescence staining.

Auxiliary splicing factor gene	Function of auxiliary splicing factor	iPSC RNA-Seq		iNCC RNA-Seq	
		Differential expression	Differential splicing	Differential expression	Differential splicing
SRSF1	Constitutive and alternative splicing activator, and has a role in EMT progression	No	No	No	No
SRSF2	Constitutive and alternative splicing activator	No	No	No	No
SRSF3	Constitutive and alternative splicing activator, and has a role in EMT progression	No	No	No	No
SRSF4	Constitutive and alternative splicing activator	No	No	No	No
SRSF5	Constitutive and alternative splicing activator	No	No	No	No
SRSF6	Constitutive and alternative splicing activator	No	No	No	No
SRSF7	Constitutive and alternative splicing activator	No	No	No	No
SRSF11	Alternative splicing repressor	No	No	No	No
SRSF9	Constitutive and alternative splicing regulator	No	No	No	No
SRSF10	General splicing repressor	No	No	No	No

TRA2A	Splicing activator	No	No	No	No
TRA2B	Splicing activator	No	No	No	No
RNPS1	Constitutive and alternative splicing regulator	No	No	No	No
SRSF12	Negative regulator of alternative splicing	No	No	No	No
U2AF1	Constitutive splicing factor	Yes (expression higher in pooled controls than patients)	No	No	No
U2AF2	Constitutive splicing factor	No	No	No	No
SNRNP70	Constitutive splicing factor	No	No	No	No
AKAP17A	Alternative splicing regulator	No	No	No	No
SRSF8	Constitutive and alternative splicing regulator	No	No	Yes (expression higher in patients than pooled controls)	No
ZRSR2	Splicing factor	No	No	No	No
RBM39	Alternative splicing regulator	No	No	No	No
CLASRP	Alternative splicing regulator	No	No	No	No
PNN	Alternative splicing regulator	No	No	No	No
SCAF11	Splicing factor	No	No	No	No
ZRANB2	Alternative splicing regulator	No	No	No	No

SRRM1	Constitutive and alternative splicing co-activator	No	No	No	No
SRRM2	Constitutive and alternative splicing co-activator	No	No	Yes (expression higher in pooled controls than patients)	Yes (increased SE in patient compared to pooled controls)
ESRP-1	Promotes epithelial cell-specific splicing pattern; downregulated during EMT	No	No	Yes (expression higher in patients than pooled controls)	No
ESRP-2	Promotes epithelial cell-specific splicing pattern; downregulated during EMT	No	No	No	Yes (increased SE in patient compared to pooled controls)
RBFOX2	Promotes mesenchymal splicing patterns for many transcripts that undergo AS during the EMT	No	No	No	Yes (increased SE in patient compared to pooled controls and MXE events)
RBM47	Regulates splicing of numerous transcripts that switch during EMT; expression downregulated during EMT	No	No	No	No
QKI	Broadly promotes mesenchymal	No	No	No	No

	splicing patterns				
MBNL1	Implicated in AS during EMT	No	No	No	Yes (increased SE in patient compared to pooled controls)
PTBP1	Implicated in AS during EMT	No	No	No	No

S3 Table: Differential expression and differential splicing of auxiliary and alternative splicing factors, including splicing factors important in the epithelial-to-mesenchymal transition (EMT), in patient iPSCs or iNCCs compared to pooled maternal and unrelated control (pooled control) iPSCs or iNCCs, as determined from RNA-Seq data. SE = skipped exon, MXE = mutually exclusive exons, AS = alternative splicing.

	**MXE**<br>80	**RI**<br>67	**SE**<br>882	**A3SS**<br>92
**A5SS**<br>83	6	6	13	2
**MXE**<br>80		4	48	6
**RI**<br>67			11	6
**SE**<br>882				18

S4 Table: Number of genes showing more than one form of alternative splicing events between pooled patient and pooled mother and unrelated control iPSCs obtained using rMATS. A3SS = alternative 3' splice site, A5SS = alternative 5'ss, MXE = mutually exclusive exons, RI =retained intron, SE = skipped exon.

Gene	Number of types of mis-splicing	Classes of mis-splicing present
SNHG14	4	MXE, RI, SE, A3SS
AC026412.1	3	A5SS, MXE, SE
AC104257.1	3	A5SS, MXE, SE
AC239809.3	3	MXE, SE, A3SS
FOXP2	3	MXE, SE, A3SS
HPN	3	A5SS, MXE, SE
PCBP1-AS1	3	A5SS, MXE, SE
SNHG17	3	A5SS, MXE, SE
TNNT1	3	MXE, SE, A3SS
ZDHHC4	3	A5SS, MXE, SE
ABCC1	2	MXE, SE
ADARB1	2	A5SS, SE
AGAP4	2	MXE, SE
ANAPC5	2	RI, A3SS
ANKMY1	2	MXE, SE
ARIH2	2	SE, A3SS
ATXN2L	2	RI, A3SS
AURKA	2	MXE, SE
B3GNTL1	2	MXE, SE
CARS2	2	A5SS, SE
CCDC15	2	MXE, SE
CCDC18-AS1	2	MXE, SE
CCDC30	2	MXE, SE
CD44	2	SE, A3SS
CFAP298	2	RI, SE
CHRD	2	A5SS, RI
CLEC7A	2	A5SS, SE
CNOT2	2	MXE, SE
COA1	2	MXE, SE
COPE	2	MXE, SE
CPNE1	2	A5SS, A3SS
DCXR	2	SE, A3SS
DDR1	2	MXE, SE
DDX11	2	RI, A3SS
DHRS12	2	A5SS, A3SS
DMAC2	2	RI, SE
DMPK	2	RI, SE

DNAH14	2	MXE, SE
DNMT1	2	MXE, RI
DRAM2	2	MXE, SE
EPN2	2	SE, A3SS
ERN2	2	RI, SE
FMR1-AS1	2	A5SS, RI
HLA-A	2	MXE, RI
IDH3G	2	MXE, SE
LARP4	2	MXE, A3SS
LINC01529	2	RI, SE
LINC01535	2	SE, A3SS
LPAR4	2	MXE, SE
LPP	2	MXE, SE
LTK	2	A5SS, RI
MARK3	2	MXE, SE
MIB1	2	MXE, SE
MLF1	2	MXE, SE
MLST8	2	RI, A3SS
MRPS15	2	MXE, SE
MRVI1	2	SE, A3SS
MTX2	2	MXE, SE
MYEOV	2	RI, SE
MYO7A	2	RI, SE
NDUFA3	2	MXE, SE
NEPRO	2	A5SS, SE
NMRK1	2	RI, SE
PCAT1	2	MXE, SE
PDE2A	2	MXE, SE
PIGQ	2	SE, A3SS
PIGT	2	SE, A3SS
PLD3	2	A5SS, SE
PRMT5	2	A5SS, RI
PSMA4	2	RI, A3SS
PSMD13	2	SE, A3SS
PSPH	2	SE, A3SS
RPL17	2	MXE, RI
SATB1-AS1	2	MXE, SE
SCART1	2	A5SS, RI
SEC24C	2	MXE, SE
SLC14A1	2	MXE, A3SS
SLC30A6	2	MXE, SE
SLC38A9	2	SE, A3SS

SLC3A2	2	MXE, SE
SRD5A3-AS1	2	MXE, SE
SS18	2	SE, A3SS
ST6GALNAC6	2	A5SS, SE
STARD5	2	RI, SE
TMEM126B	2	MXE, SE
TMEM14B	2	MXE, SE
TSC1	2	SE, A3SS
TSFM	2	A5SS, RI
TSPAN17	2	A5SS, SE
TSSC2	2	MXE, SE
TULP3	2	MXE, SE
TVP23A	2	MXE, SE
WDR97	2	SE, A3SS
ZNF185	2	MXE, SE
ZNF232	2	MXE, SE
ZSCAN10	2	MXE, SE
ZSWIM7	2	RI, SE

S5 Table: Identities of genes showing multiple classes of significantly altered splicing events in pooled patient iPSCs compared to pooled mother and unrelated control iPSCs. A5SS = alternative 5’ splice site, A3SS = alternative 3’ splice site, MXE = mutually exclusive exons, RI = retained intron, SE = skipped exon.

	**A5SS**<br>194	**MXE**<br>155	**RI**<br>172	**SE**<br>1619
**A3SS**<br>155	7	5	10	40
**A5SS**<br>194		9	12	65
**MXE**<br>155			7	98
**RI**<br>172				43

S6 Table: Number of genes showing more than one form of alternative splicing events between pooled patient iNCCs and pooled mother and unrelated control iNCCs obtained using rMATS. A3SS = alternative 3' splice site, A5SS = alternative 5'ss, MXE = mutually exclusive exons, RI = retained intron, SE = skipped exon.

Gene	Number of different types of mis-splicing	Types of mis-splicing present
COMMD4	4	A5SS, MXE, RI, SE
ADAM15	3	A5SS, MXE, SE
AKAP8L	3	A5SS, RI, SE
CCHCR1	3	A3SS, A5SS, SE
CPNE1	3	A3SS, A5SS, SE
CTNND1	3	A5SS, MXE, SE
DMTF1	3	A3SS, MXE, SE
EPB41	3	A5SS, MXE, SE
EPB41L3	3	MXE, RI, SE
FAM122C	3	MXE, RI, SE
GGT1	3	A3SS, RI, SE
HDAC7	3	A5SS, RI, SE
LUCAT1	3	A5SS, RI, SE
NAP1L4	3	A3SS, MXE, SE
PCBP1-AS1	3	A3SS, MXE, SE
PFKM	3	A5SS, MXE, SE
PLD3	3	A3SS, A5SS, SE
RHOT2	3	A5SS, RI, SE
RPS24	3	MXE, RI, SE
SGCE	3	A3SS, RI, SE
SNHG14	3	A3SS, RI, SE
SNHG17	3	A5SS, MXE, SE
SPG7	3	A5SS, RI, SE
SUGP2	3	A3SS, RI, SE
TPM2	3	MXE, RI, SE
ZHX3	3	A5SS, MXE, SE
ZSWIM7	3	MXE, RI, SE
ABCB8	2	MXE, SE
AC002074.1	2	MXE, SE
AC012651.1	2	RI, SE
AC026412.1	2	A5SS, SE
AC068831.7	2	A3SS, SE
ACCS	2	A5SS, SE
ACTN1	2	MXE, SE
ACYP1	2	MXE, SE
ADGRB2	2	MXE, SE
AFDN	2	A3SS, SE
AGRN	2	MXE, SE
AKAP3	2	A5SS, SE

AMHR2	2	A5SS, RI
ANK2	2	A3SS, SE
ANKDD1A	2	A5SS, SE
ANKZF1	2	A5SS, RI
AP1G1	2	RI, SE
AP1G2	2	A3SS, SE
APBB3	2	A3SS, SE
ARHGEF40	2	RI, SE
ARHGEF9	2	MXE, SE
ASL	2	MXE, SE
ATP13A2	2	A3SS, A5SS
ATXN2L	2	A3SS, RI
AURKA	2	MXE, SE
BCS1L	2	A5SS, SE
BECN1	2	A5SS, SE
BRCA1	2	A5SS, SE
CACNA1E	2	MXE, SE
CAPN7	2	MXE, SE
CARF	2	A5SS, MXE
CCDC15	2	MXE, SE
CCDC173	2	A3SS, SE
CCNB1IP1	2	MXE, SE
CCNT1	2	MXE, SE
CD44	2	A3SS, SE
CDK10	2	RI, SE
CDK5RAP3	2	A5SS, RI
CERS5	2	A3SS, A5SS
CHD2	2	MXE, SE
CIB2	2	A3SS, SE
CLCN6	2	A3SS, SE
CNOT2	2	MXE, SE
COA1	2	MXE, SE
COL9A3	2	A3SS, SE
CPAMD8	2	A5SS, SE
CRELD1	2	A5SS, RI
CREM	2	MXE, SE
CROCCP2	2	A3SS, SE
CSNK1D	2	RI, SE
CYP3A5	2	A3SS, SE
DCUN1D4	2	A5SS, SE
DEDD2	2	MXE, SE
DGLUCY	2	A5SS, SE

DHRS4L2	2	RI, SE
DMAC2	2	RI, SE
DMKN	2	RI, SE
DNAH14	2	MXE, SE
DNAJC10	2	A3SS, MXE
DNM1	2	A3SS, SE
DNM2	2	MXE, SE
DNMT1	2	MXE, RI
DPY19L3	2	RI, SE
DRAM2	2	MXE, SE
ECSIT	2	A5SS, SE
EFCAB12	2	RI, SE
EFNA1	2	A3SS, SE
EIF4A2	2	MXE, SE
ELMOD3	2	MXE, SE
ELP2	2	MXE, SE
EML4	2	MXE, SE
EXOSC8	2	RI, SE
FAM135A	2	A3SS, SE
FAM219B	2	RI, SE
FAM241B	2	RI, SE
FAM66C	2	A5SS, SE
FES	2	A5SS, SE
FGFR1	2	MXE, SE
FGFR2	2	MXE, SE
FGFR3	2	MXE, SE
GATAD2A	2	A3SS, SE
GORASP1	2	A3SS, SE
GTF2E2	2	MXE, SE
HACL1	2	MXE, SE
HDAC2-AS2	2	A5SS, SE
HERC2P9	2	A5SS, SE
HLCS	2	MXE, SE
HNRNPH1	2	A5SS, RI
HOOK3	2	MXE, SE
IMMP1L	2	A5SS, SE
ING4	2	A5SS, SE
INTS6L	2	RI, SE
IRF3	2	A5SS, SE
IVNS1ABP	2	MXE, SE
KIF23	2	MXE, SE
KLC1	2	A5SS, SE

L3MBTL2	2	A5SS, SE
LARGE2	2	A3SS, RI
LIMS2	2	RI, SE
LINC01198	2	A5SS, SE
LPP	2	MXE, SE
LRRC77P	2	MXE, SE
LYPD6B	2	MXE, SE
MAMDC4	2	RI, SE
MAP4K4	2	A5SS, SE
MATR3	2	MXE, SE
METTL23	2	A5SS, SE
MIB1	2	MXE, SE
MITD1	2	RI, SE
MLH1	2	A3SS, SE
MLST8	2	A3SS, RI
MLXIPL	2	A3SS, A5SS
MRPS15	2	MXE, SE
MSRB3	2	MXE, SE
MTX2	2	MXE, SE
MUS81	2	A3SS, RI
MYH10	2	MXE, SE
NAP1L1	2	MXE, SE
NBN	2	A3SS, SE
NEMP1	2	A5SS, SE
NGLY1	2	MXE, SE
NT5C2	2	A5SS, SE
OGT	2	A3SS, SE
PC	2	A5SS, SE
PCAT1	2	MXE, SE
PHPT1	2	RI, SE
PHYKPL	2	A3SS, SE
PICALM	2	MXE, SE
PIGG	2	A5SS, SE
PLAA	2	MXE, SE
PNISR	2	A5SS, SE
POLR2G	2	A5SS, SE
PPP1R12C	2	RI, SE
PRMT7	2	MXE, SE
PRPSAP2	2	MXE, SE
PTCH1	2	A5SS, SE
PTK2	2	A5SS, SE
PUS1	2	MXE, SE

PVT1	2	A5SS, SE
PWRN1	2	MXE, SE
RAB27A	2	A5SS, SE
RBFOX1	2	A5SS, SE
RBFOX2	2	MXE, SE
RHOC	2	A5SS, SE
RHOT1	2	MXE, SE
RIPOR1	2	RI, SE
RNF167	2	RI, SE
RNF181	2	A3SS, SE
RPL17	2	RI, SE
RPRD1A	2	MXE, SE
RPS9	2	RI, SE
RSRP1	2	RI, SE
SAP30BP	2	RI, SE
SCARB1	2	RI, SE
SEC31A	2	MXE, SE
SEPT2	2	A5SS, MXE
SETD5	2	MXE, SE
SGO1	2	A5SS, SE
SH2B1	2	A5SS, SE
SH3D19	2	MXE, SE
SH3YL1	2	A3SS, SE
SLC25A19	2	A5SS, SE
SLC3A2	2	MXE, SE
SLC50A1	2	A5SS, SE
SMG7	2	MXE, SE
SMPD4	2	A5SS, SE
SMUG1	2	MXE, SE
SNTG2	2	MXE, SE
SPTY2D1OS	2	A5SS, SE
SS18	2	A3SS, SE
ST3GAL1	2	MXE, SE
ST6GALNAC6	2	A5SS, SE
STK33	2	A5SS, SE
STRA6LP	2	A5SS, RI
SULF2	2	RI, SE
SUN1	2	MXE, SE
TAF1	2	A5SS, SE
TANGO2	2	MXE, SE
TBC1D1	2	MXE, SE
TCTN1	2	A3SS, SE

TIAL1	2	A3SS, SE
TLE4	2	MXE, SE
TM7SF3	2	MXE, SE
TMEM126B	2	MXE, SE
TMEM134	2	A3SS, A5SS
TMEM139	2	A3SS, SE
TMEM191C	2	A3SS, RI
TMEM91	2	A3SS, RI
TNNT3	2	A3SS, SE
TNRC6A	2	MXE, SE
TPD52L2	2	MXE, SE
TRMT1	2	A5SS, SE
TRMU	2	A3SS, SE
TTN-AS1	2	MXE, SE
TVP23C-CDRT4	2	MXE, SE
UBE2F	2	MXE, SE
UBE2V1	2	A5SS, SE
UNC80	2	A5SS, SE
VPS13B	2	MXE, SE
VPS29	2	MXE, SE
WDR27	2	MXE, SE
ZBED3-AS1	2	MXE, SE
ZBTB8OS	2	MXE, SE
ZFAND4	2	A5SS, SE
ZMAT1	2	A3SS, SE
ZNF10	2	RI, SE
ZNF107	2	MXE, SE
ZNF185	2	MXE, SE
ZNF266	2	A5SS, SE
ZNF561	2	MXE, SE
ZNF692	2	A5SS, SE
ZWILCH	2	A3SS, MXE

S7 Table: Identities of genes showing multiple classes of significantly altered splicing events in pooled patient iNCCs compared to pooled mother and unrelated control iNCCs. A5SS = alternative 5' splice site, A3SS = alternative 3' splice site, MXE = mutually exclusive exons, RI = retained intron, SE = skipped exon.
